# Behavioural and Genetic Predictors of Positive Symptoms in Psychotic Disorders

**DOI:** 10.1192/j.eurpsy.2026.10526

**Published:** 2026-07-31

**Authors:** D. C. Godoi, J. T. Walters, S. E. Legge

**Affiliations:** 1 School of Psychology; 2 Centre for Neuropsychiatric Genetics and Genomics, Division of Psychological Medicine and Clinical Neurosciences, School of Medicine, Cardiff University; 3 Centre for Neuropsychiatric Genetics and Genomics, Division of Psychological Medicine and Clinical Neurosciences, School of Medicine, Cardiff University, Cardiff, United Kingdom

## Abstract

**Introduction:**

Schizophrenia and related psychotic disorders are characterised by positive, negative and disorganised symptoms. Inconsistent associations have been reported between clinical and social predictors of psychosis and positive symptom severity, perhaps due to positive symptoms often being considered as a single construct. Disentangling the heterogeneity of positive symptoms could give insight into symptom-specific predictors, and the mechanisms underlying psychosis.

**Objectives:**

To investigate the association between clinical, social and genetic predictors of psychosis and the severity of individual psychotic symptoms.

**Methods:**

1297 individuals with an ICD-10 diagnosis of schizophrenia or a related psychotic disorder were included from a cross-sectional clinical sample called CardiffCOGS. Lifetime symptom severity was measured using the Scale for Assessment of Positive Symptoms (SAPS). Clinical and social predictors were assessed through clinical interviews and validated questionnaires. Univariate linear regressions with age and sex as covariates were used to test whether each predictor was associated with individual positive symptoms. Polygenic Risk Scores (PRS) for schizophrenia, bipolar, ADHD, autism, and intelligence were calculated and tested for association with positive symptoms. To account for multiple testing, the false-discovery rate was controlled using the Benjamini-Hochberg method.

**Results:**

Earlier age of onset, cannabis use and tobacco smoking were each associated with higher severity of multiple hallucinations and delusions. Premorbid IQ was significantly associated with higher severity of religious (β= 0.1706, p= 9.51x10^-5^), grandiose (β= 0.144, p= 1.40x10^-7^), referential (β= 0.128, p= 0.02) and guilt-based (β= 0.089, p= 0.02) delusions, but negatively associated with global (β= -0.167, p= 2.44x10^-3^) and auditory hallucinations (β= -0.253, p= 8.28x10^-3^). A similar pattern of findings was found for educational attainment. Childhood abuse was associated with severity of auditory (β= 0.088, p= 1.92x10^-3^), visual (β= 0.080, p= 6.55x10^-4^) and somatosensory (β = 0.042, p= 0.04) hallucinations but had no association with delusions. The only significant PRS association was between intelligence PRS and religious delusions (β= 0.252, p=4.61x10^-3^).

**Conclusions:**

Earlier age of onset, cannabis use and tobacco smoking were found to be general predictors of severity of positive symptoms. Premorbid IQ and educational attainment may enhance delusion-formation while dampening the severity, or reporting of hallucinations. Meanwhile, childhood abuse showed a specific effect on severity of hallucinations. Genetic liability to schizophrenia or other mental health conditions do not strongly predict the severity of psychosis in those with psychotic disorders. These findings align with current models of psychosis, and reveal factors underlying the heterogeneity of symptoms in psychosis.

**Disclosure of Interest:**

None Declared

